# High-dimensional quantum key distribution implemented with biphotons

**DOI:** 10.1038/s41598-023-28382-w

**Published:** 2023-01-21

**Authors:** Comfort Sekga, Mhlambululi Mafu, Makhamisa Senekane

**Affiliations:** 1 Department of Physics and Astronomy, Botswana International University of Science and Technology, P/Bag 16, Palapye, Botswana; 2 Department of Physics, Case Western Reserve University, Cleveland, OH 44106 USA; 3 Institute for Intelligent Systems, University of Johannesburg, Johannesburg, 2006 South Africa; 4 National Institute for Theoretical and Computational Sciences, Gauteng, 2006 South Africa

**Keywords:** Quantum information, Information theory and computation, Optics and photonics

## Abstract

We present a high-dimensional measurement device-independent (MDI) quantum key distribution (QKD) protocol employing biphotons to encode information. We exploit the biphotons as qutrits to improve the tolerance to error rate. Qutrits have a larger quantum system; hence they carry more bits of classical information and have improved robustness against eavesdropping compared to qubits. Notably, our proposed protocol is independent of measurement devices, thus eliminating the possibility of side-channel attacks. Also, we employ the finite key analysis approach to study the performance of our proposed protocol under realistic conditions where finite resources are used. Furthermore, we simulated the secret key rate for the proposed protocol in terms of the transmission distance for different fixed amounts of signals. The results prove that this protocol achieves a considerable secret key rate for a moderate transmission distance of 90 km by using $10^{16}$ signals. Moreover, the expected secret key rate was simulated to examine our protocol’s performance at various intrinsic error rate values, $Q=(0.3\%,0.6\%,1\%)$ caused by misalignment and instability due to the optical system. These results show that reasonable key rates are achieved with a minimum data size of about $10^{14}$ signals which are realizable with the current technology. Thus, implementing MDI-QKD using finite resources while allowing intrinsic errors due to the optical system makes a giant step forward toward realizing practical QKD implementations.

## Introduction

Quantum key distribution (QKD) is a procedure for establishing symmetric cryptographic keys between legitimate participants by distributing quantum states^1^. In principle, QKD provides information-theoretic security, guaranteed by quantum mechanical laws^2^. Notably, QKD has developed from mere theoretical security proofs to commercial applications over the past two decades. However, practical QKD has yet to attain its full deployment owing to security lapses in the theoretical security proofs that arise from certain assumptions about the sources or devices belonging to Alice and Bob^3^. For example, QKD protocols depend on trusted device scenarios, i.e., it is assumed that no information is leaked from the transmitters or the senders, which is very challenging to guarantee in practice. This creates a gap between theory and experimental implementations, opens loopholes, and leads to various possible attacks on the QKD systems^4^. Moreover, during implementations, QKD protocols depend on trusted device scenarios, and this assumption allows the protocols to achieve effective rates. Unfortunately, this provides an opportunity for harmful attacks, such as the side-channel attacks^5^. As a result, besides the enormous theoretical and experimental quantum cryptography progress, some work remains before fully deploying QKD in commercial applications. Hence, the device-independent (DI) QKD provides an improved security degree compared to conventional QKD schemes by lessening the number of assumptions required concerning the physical devices used^6^.

The security of the DI-QKD depends on the violation of Bell inequalities^7^. However, the DI-QKD needs the loophole-free Bell experiments, making it impossible to realize using existing technologies. Recent demonstrations of various attacks highlight this on practical QKD systems^5^. As a result, the measurement device-independent (MDI) QKD provides an improved practical solution intrinsically insensitive to entire attacks caused by side channels. These attacks target a measurement device and remove the detection-associated security loopholes^8,9^. Furthermore, the participating parties are connected by an untrusted relay for an MDI-QKD, leading to a considerable gain in transmission distances compared to the traditional QKD schemes. Thus, this makes these set-ups ideally suitable for quantum networks. Moreover, some experimental demonstrations using MDI-QKD have been conducted in Refs.^10,11^. However, the practical MDI-QKD still experiences relatively low key rates compared to the conventional BB84 protocol due to the requirement of Bell-state measurements. Apart from these advances, adopting QKD widely has been a challenge, and it has been demonstrated that large-scale deployment will likely require chip-based devices for improved performance, miniaturization, and enhanced functionality^12–18^. Most significantly, these integrated photonic chips offer numerous benefits such as low cost, low power consumption, and well-established batch fabrication techniques^19^.

To encode information in a QKD protocol, the parties must choose a certain degree of freedom, for instance, polarization or phase properties of single-photon as quantum states^1,20^. This means one classical bit of information is encoded onto each quantum state, resulting in limited secret key rates^21^. As a result, high dimensional encoding presents a promising solution to address the limitation of low secret key rates in QKD^22^. High dimensional quantum systems allow the communicating parties in QKD to encode information beyond one bit per signal^23^. The secret key rate, which may be limited by inevitable factors such as losses in the channel, and source and detector flaws, can be significantly improved by high-dimensional encoding where each photon can encode up to ${\text {log}}_{2}d > 1$ bits. This allows a considerable amount of information to be sent in a given transmission of the signal in the channel. Notably, previous studies indicate that the resistance to noise of the protocols increases when one increases the dimension, both for one-way^24–26^ and two-way post-processing^27^. Furthermore, compared to qubit operations, high dimensional quantum states are robust against noise due to the background and hacking attacks^24^.

Qudits have been proven to be robust against quantum cloning compared to their qubit counterparts^28^. Thus, they are an excellent illustration of the effectiveness of high-dimensional quantum systems since they lead to higher error thresholds making it challenging for the eavesdropper to intercept a high-dimensional QKD scheme. Owing to this, the merits of several degrees of freedom have been examined for high dimensional QKD, which includes position-momentum^29^, orbital angular momentum^23,30–33^ and time energy^34–41^ and MDI-QKDs employing high-dimensional quantum states^42–44^. Another approach to realizing high dimensional encoding is using biphotons corresponding to a pair of indistinguishable photons with qutrit (i.e., a three-level quantum system) representation. Biphotons or pairs of entangled photons form a two-photon light and constitute one of the most critical states of light in recent quantum information and quantum optics^45,46^. The generation, manipulation, and detection procedures for single-mode biphoton beams with linear optics have been demonstrated in Refs.^47–49^.

We propose an MDI-QKD protocol that encodes information on qutrit states by exploiting the polarization state of single-mode biphoton field. Using biphotons as qutrits enhances the attainable secret key rate and security due to improved information capacity per photon and the high noise tolerance^45^. To examine the practicality of the proposed protocol, we investigate the finite-key bounds against the general attacks based on entropic uncertainty relations. Moreover, this security analysis pertains to the implementation using the decoy states. This enables the proposed protocol to be secure against Photon-Number-Splitting (PNS) attacks^1^. For the finite-key study, the statistical fluctuations are catered by leveraging the large deviation theory, particularly the multiplicative Chernoff bound^50^. This bound provides the tightest bounds on estimated parameters for the high-loss regime. More recently, a similar work on three-dimensional MDI-QKD was proposed by Jo et al.^51^. Their proposed protocol exploits the time bin entangled qutrit states to encode information and employs a tripartite qutrit discrimination setup. The setup relies on a tritter and non-destructive photon number measurements to filter the states for Bell state measurements. Conversely, our proposed MDI-QKD protocol utilizes the Mach Zehnder interferometer to generate biphoton states and the Brown Twiss schemes to achieve Bell state measurements. While the scheme in Ref.^51^ is more efficient in terms of fewer resources used in Charlie’s measurement site; it involves non-destructive measurements, which may open up a possibility of side-channel attacks. Another noticeable difference is that our work considers finite key analysis and studies the performance of the qutrit MDI-QKD under realistic conditions by determining the key rate as a function of transmission distance. The work in Ref.^51^ only provides tolerable error bounds that allow one to distill a secure key. Therefore, apart from this introduction, the following section describes the proposed protocol, while the next section provides the security proof based on the entropic uncertainty principle. After that, we simulate the performance of our protocol to demonstrate its feasibility and conclude this paper.

## Protocol definition

### Biphotons

The pure polarization state for a single-mode biphoton field is expressed according to the following^45,47^:

1 $$\begin{aligned} |\Phi \rangle =c_{1}|2,0\rangle +c_{2}|1,1\rangle +c_{3}|0,2\rangle , \end{aligned}$$

where $c_{i}=|c_{i}|e^{i\phi _{i}}$ are complex amplitudes. The notation $|n_{h},n_{v}\rangle$ represents a state that consists of *n* photons in the horizontal (*h*) mode as well as *n* photons in the vertical (*v*) polarization mode. Therefore, from Eq. (1), we can observe that a biphoton has a three-level quantum system (qutrit) representation, hence its use for ternary quantum information encoding. To realize the QKD protocol, one needs at least two mutually unbiased bases (MUB) from available $d+1$ MUBs. The two orthonormal bases ${\mathscr {M}}_{1}=|\phi \rangle _{i}$, where $i \in \{0,1,2\}$ and ${\mathscr {M}}_{2}=|\Phi \rangle _{j}$, where $j \in \{0,1,2\}$ for a 3-dimensional Hilbert space ${\mathscr {H}}_{3}$ are considered mutually unbiased when all pairs of basis vectors $|\phi \rangle _{i}$ and $|\Phi \rangle _{j}$ satisfies

2 $$\begin{aligned} |\langle \phi _{i}|\Phi _{j} \rangle |^{2}=\frac{1}{3}. \end{aligned}$$

For biphotons, the standard basis is expressed in terms of the orthonormal states $|\alpha \rangle =|2,0\rangle$, $|\beta \rangle =|0,2\rangle$ and $|\gamma \rangle =|1,1\rangle$. The states $|2,0\rangle$ and $|0,2\rangle$ represent type-I phase matching, while $|1,1\rangle$ corresponds to type II phase matching, and one can obtain these biphoton fields through spontaneous parametric down-conversion (SPDC). The other three MUBs are realized from the superposition of the basis vectors as follows

3 $$\begin{aligned} |\acute{\alpha }\rangle&=\frac{1}{\sqrt{3}}(|\alpha \rangle +|\beta \rangle +|\gamma \rangle )\end{aligned}$$

4 $$\begin{aligned} |\acute{\beta }\rangle&=\frac{1}{\sqrt{3}}(|\alpha \rangle +\omega |\beta \rangle +\omega ^{2}|\gamma \rangle )\end{aligned}$$

5 $$\begin{aligned} |\acute{\gamma }\rangle&=\frac{1}{\sqrt{3}}(|\alpha \rangle +\omega ^{2}|\beta \rangle +\omega |\gamma \rangle ), \end{aligned}$$

6 $$\begin{aligned} |\bar{\alpha }\rangle&=\frac{1}{\sqrt{3}}(|\alpha \rangle +|\beta \rangle +\omega |\gamma \rangle )\end{aligned}$$

7 $$\begin{aligned} |\bar{\beta }\rangle&=\frac{1}{\sqrt{3}}(|\alpha \rangle +\omega |\beta \rangle +|\gamma \rangle )\end{aligned}$$

8 $$\begin{aligned} |\bar{\gamma }\rangle&=\frac{1}{\sqrt{3}}(\omega | \alpha \rangle +|\beta \rangle +|\gamma \rangle ), \end{aligned}$$

and

9 $$\begin{aligned} |\tilde{\alpha }\rangle&=\frac{1}{\sqrt{3}}(|\alpha \rangle +|\beta \rangle +\omega ^{2}|\gamma \rangle )\end{aligned}$$

10 $$\begin{aligned} |\tilde{\beta }\rangle&=\frac{1}{\sqrt{3}}(|\alpha \rangle +\omega ^{2}|\beta \rangle +|\gamma \rangle )\end{aligned}$$

11 $$\begin{aligned} |\tilde{\gamma }\rangle&=\frac{1}{\sqrt{3}}(\omega ^{2}|\alpha \rangle +|\beta \rangle +|\gamma \rangle ), \end{aligned}$$

where $\omega =\exp (i2\pi /2)$. In our scenario, to realize high dimensional encoding with biphotons, we propose an MDI-QKD protocol exploiting two Fourier transformed bases, ${\mathscr {M}}_{1}=\{|\acute{\alpha }\rangle ,|\acute{\beta }\rangle ,|\acute{\gamma }\rangle \}$ and ${\mathscr {M}}_{2}=\{|\bar{\alpha }\rangle ,|\bar{\beta }\rangle ,|\bar{\gamma }\rangle \}$.

### Preparation of states

**Figure 1:**
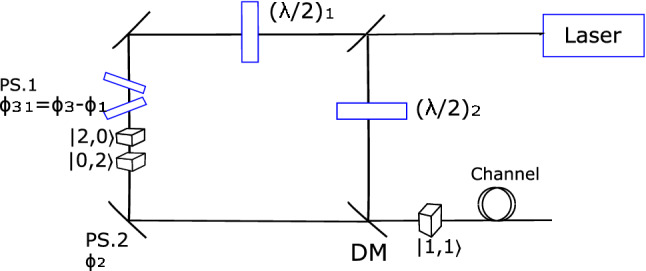
Mach–Zehnder interferometer set up used by Alice and Bob for biphoton state preparation. The laser beam is pumped towards a non-symmetric beam-splitter that transmits 2/3 and reflects 1/3 of the beam through the long and short arms. The $(\lambda /2)_{i}$ represents the halve wave plates for manipulating the amplitude, $c_{i}$ of the states. The phase shifters (PS. 1 and PS. 2) introduce relative phases between the states. The DM denotes the dichroic mirror, which transmits two biphotons created using type I non-linear crystals from the long arm of the interferometer and reflect the pump from the short arm towards the type II non-linear crystal for the creation of state $|1,1\rangle$.

Alice (Bob) starts by randomly preparing qutrit states from two mutually unbiased bases ${\mathscr {M}}_{1}=\{|\acute{\alpha }\rangle ,|\acute{\beta }\rangle ,|\acute{\gamma }\rangle \}$ and ${\mathscr {M}}_{2}=\{|\bar{\alpha }\rangle ,|\bar{\beta }\rangle ,|\bar{\gamma }\rangle \}$. The biphoton states $\{|\acute{\alpha }\rangle ,|\bar{\alpha }\rangle \}$, $\{|\acute{\beta }\rangle ,|\bar{\beta }\rangle \}$ and $\{|\acute{\gamma }\rangle ,|\bar{\gamma }\rangle \}$ are assigned bit values 0, 1 and 2, (the value 2 is converted to binary digit during sifting to obtain two bits per signal) respectively. The states are prepared using a Mach–Zehnder interferometer consisting of 3 arms and appropriate non-linear crystals in each arm. This is illustrated in Fig. 1.

The laser beam is pumped onto a non-symmetric beamsplitter that transmits two-thirds of the beam through the interferometer’s long arm and reflects the other beam via the short arm. The beam in the long arm is pumped towards the type I non-linear crystals for generating states $|2,0\rangle$ and $|0,2\rangle$ resulting in a superposition

12 $$\begin{aligned} |\Phi \rangle =c_{1}|2,0\rangle +e^{i\phi _{31}} c_{3}|0,2\rangle , \end{aligned}$$

with $e^{i\phi _{31}}$ representing the relative phase between the states realized through phase shifters. The half-wave plate is used for manipulating the amplitudes of these states. A cut-off flitter is employed to remove the pump. After passing through the filter, the states arrive at the piezoelectric translator, where the phase shift, $\phi _{2}$, is introduced between the superposition of states defined in Eq. (12) and the state $|1,1\rangle$. The reflected beam traveling through the short arm is guided towards the half-wave plate to control the amplitude corresponding to the state $|1,1\rangle$. The pump is reflected at the dichroic mirror towards the type II non-linear crystals to create the state $|1,1\rangle$. The type I biphotons from the long arm of the interferometer is transmitted via the dichroic mirror. Thus, at the interferometer’s output, we have the superposition of three basic states in Eq. (1), which are then propagated through the insecure channel to the measurement site. Different states from two mutually unbiased bases are produced by adjusting the phase shifters and halve wave plates according to Table 1.

**Table 1:** The parameter settings for biphoton states from two mutually unbiased bases used in our QKD scheme.

State	$|c_{1}|$	$|c_{2}|$	$|c_{3}|$	$\phi _{1}$	$\phi _{2}$	$\phi _{3}$
$|\acute{\alpha }\rangle$	$\frac{1}{\sqrt{3}}$	$\frac{1}{\sqrt{3}}$	$\frac{1}{\sqrt{3}}$	0	0	0
$|\acute{\beta }\rangle$	$\frac{1}{\sqrt{3}}$	$\frac{1}{\sqrt{3}}$	$\frac{1}{\sqrt{3}}$	0	$120^{\circ }$	$-120^{\circ }$
$|\acute{\gamma }\rangle$	$\frac{1}{\sqrt{3}}$	$\frac{1}{\sqrt{3}}$	$\frac{1}{\sqrt{3}}$	0	$-120^{\circ }$	$120^{\circ }$
$|\acute{\alpha }\rangle$	$\frac{1}{\sqrt{3}}$	$\frac{1}{\sqrt{3}}$	$\frac{1}{\sqrt{3}}$	$120^{\circ }$	0	0
$|\bar{\beta }\rangle$	$\frac{1}{\sqrt{3}}$	$\frac{1}{\sqrt{3}}$	$\frac{1}{\sqrt{3}}$	0	$120^{\circ }$	0
$|\bar{\gamma }\rangle$	$\frac{1}{\sqrt{3}}$	$\frac{1}{\sqrt{3}}$	$\frac{1}{\sqrt{3}}$	0	0	$120^{\circ }$

The complex amplitudes $|c_{i}|$ are realized through the use of half-wave plates. The $\phi _{i}$ are realized by use of phase shifters.

### Measurement

**Figure 2:**
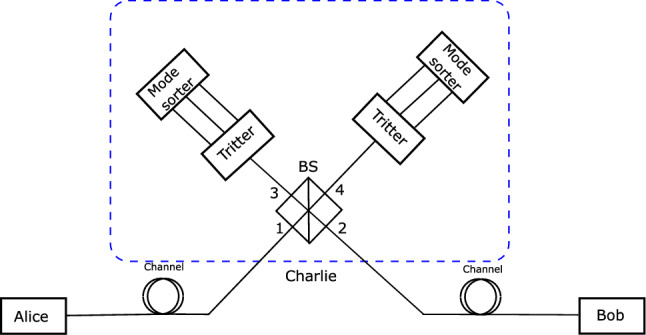
The generic measurement set up for our proposed MDI-QKD. Alice and Bob start by preparing the biphoton states from two mutually unbiased bases and send them through the unsecured channel to Charlie. Charlie allows the states to interfere in the symmetric beam-splitter (BS) upon receiving the states. The photons are directed towards the tritter and eventually detected in the Brown Twiss scheme (mode sorter).

Charlie allows the biphotons from Alice and Bob to interfere in a symmetric beam-splitter upon receiving the states. As a result, the Hong Ou Mandel effect occurs (see Fig. 2). The beam-splitter action can be described as follows. Let us assume Alice’s biphoton state at the input arm of the beam-splitter is denoted by $|\psi \rangle _{1}$, then its transformation can be described as

13 $$\begin{aligned} a^{\dagger }_{1}|\psi \rangle _{1}\rightarrow \frac{1}{\sqrt{2}}(|\psi \rangle _{3}+|\overline{\psi }\rangle _{4}). \end{aligned}$$

Similarly if Bob’s state at the input arm is $|\acute{\psi }\rangle _{2}$ then the action of the beam-splitter is described as

14 $$\begin{aligned} a_{2}^{\dagger }|\acute{\psi }\rangle _{2}\rightarrow \frac{1}{\sqrt{2}}(|\overline{\acute{\psi }}\rangle _{3}-|\acute{\psi }\rangle _{4}), \end{aligned}$$

where $a^{\dagger }_{i}$ denotes the creation operator and $|\bar{x}\rangle$ is the reflected state. The overall beam-splitter transformations are described as

15 $$\begin{aligned} |\psi \rangle _{1}|\acute{\psi }\rangle _{2}&\xrightarrow {BS}\frac{1}{\sqrt{2}}(|\psi \rangle _{3}+|\overline{\psi }\rangle _{4})\otimes \frac{1}{\sqrt{2}} (|\overline{\acute{\psi }}\rangle _{3}-|\acute{\psi }\rangle _{4}) \\&=\frac{1}{\sqrt{2}}(|\psi \rangle _{3}|\overline{\acute{\psi }}\rangle _{3}-|\psi \rangle _{3}|\acute{\psi }\rangle _{4} +|\overline{\psi }\rangle _{4}|\overline{\acute{\psi }}\rangle _{3}-|\overline{\psi }\rangle _{4}|\acute{\psi }\rangle _{4}) \\&=\frac{1}{\sqrt{2}}(|\Psi ^{+}\rangle +|\Psi ^{-}\rangle ) \end{aligned}$$

where

16 $$\begin{aligned} |\Psi ^{+}\rangle =\frac{1}{\sqrt{2}}(|\psi \rangle _{3}|\overline{\acute{\psi }}\rangle _{3} -|\overline{\psi }\rangle _{4}|\acute{\psi }\rangle _{4}) \end{aligned}$$

and

17 $$\begin{aligned} |\Psi ^{-}\rangle =\frac{1}{\sqrt{2}}(|\overline{\psi }\rangle _{4} |\overline{\acute{\psi }}\rangle _{3}-|\psi \rangle _{3}|\acute{\psi }\rangle _{4}). \end{aligned}$$

The subscripts 1, 2, and 3, 4 denote a beam-splitter’s input and output ports, respectively. According to the Hong Ou Mandel interference, identical photons will leave the beam-splitter from a similar output port, and distinguishable photons from both input ports of the beam-splitter will exit in both output ports. Let Alice and Bob choose a similar biphoton states $|\acute{\alpha }_{A}\rangle$ and $|\acute{\alpha }_{B}\rangle$, respectively. Therefore, based on the Hong Ou Mandel interference, the state $|\Psi ^{-}\rangle$ in Eq. (17) will disappear, and the resultant state is

18 $$\begin{aligned} |\Psi ^{+}\rangle =\frac{1}{\sqrt{2}}(|\acute{\alpha }_{A}\rangle _{3}|\overline{\acute{\alpha }_{B}}\rangle _{3} -|\overline{\acute{\alpha }_{B}}\rangle _{4}|\acute{\alpha }_{A}\rangle _{4}). \end{aligned}$$

**Figure 3:**
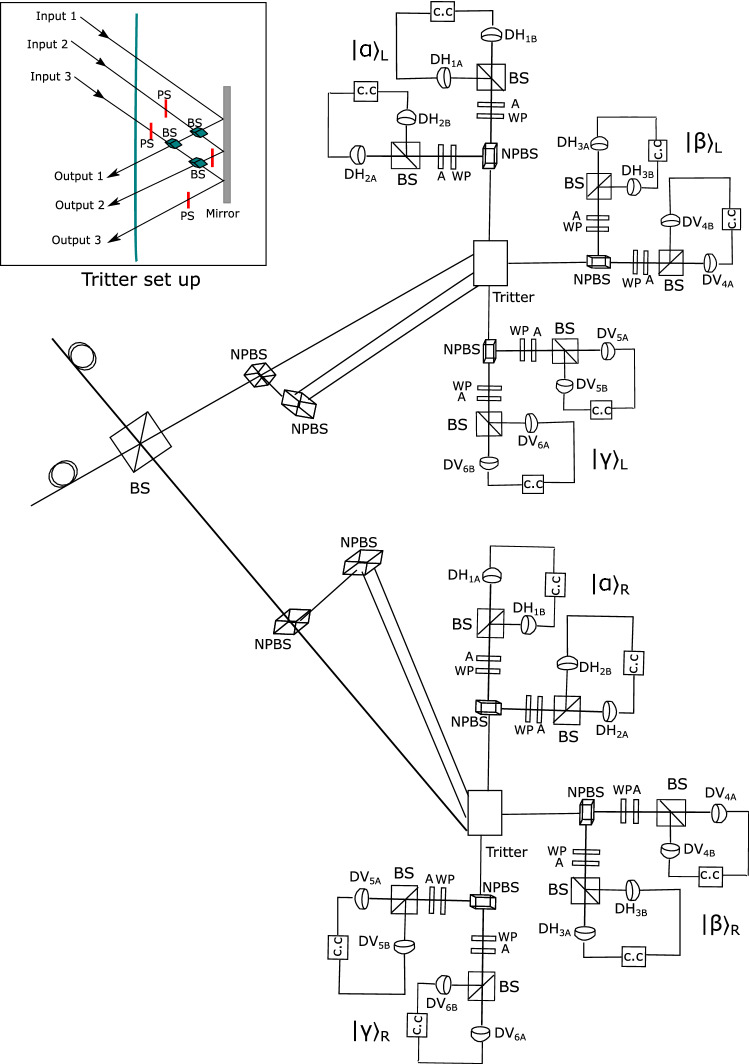
Illustration of the measurement set up at Charlie’s site. The incoming biphotons interfere in the beam-splitter (BS) and exit through either of two output ports towards the two non-symmetric beam-splitter (NPBS), where they are further split into three channels and directed towards the tritter. The tritter setup shown in the upper left is a three-input-output port splitter. It comprises three conventional beam-splitters (BS), four-phase shifters (PS), and a mirror. The output ports of the tritter are linked with three Brown Twiss schemes for detecting the three basic states $|\alpha \rangle$, $|\beta \rangle$, and $|\gamma \rangle$. Each Brown Twiss scheme consists of a non-symmetric beam-splitter (NPBS), wave plates (WP), polarization analyser (A), and detectors (D${\textbf{P}}_{i}$) where ${\textbf{P}} \in \{H,V\}$ represents polarization mode and subscript *i* denotes single photons forming biphotons. The abbreviation c.c corresponds to coincident counting or detection.

Therefore, identical biphotons will always appear in the same output arm of BS (3rd arm or 4th arm). Otherwise, if Alice and Bob prepare opposite biphotons, both $|\Psi ^{-}\rangle$ and $|\Psi ^{-}\rangle$ will exist, and there is a non-zero probability that the biphotons will exit at both output arms of the BS. The photons that exit the beam-splitter are transmitted toward the three input-output ports beam-splitter (tritter). The photons are directed toward the two non-polarizing beam-splitters to separate them into three channels from each output of the symmetric beam-splitter connected to each input of the two tritters (see Fig. 3). The probability of photons exiting through any of the output ports of the tritter is governed by the unitary matrix

19 $$\begin{aligned} {\mathscr {U}}=\frac{1}{\sqrt{3}} \begin{pmatrix} 1 &{} 1 &{} 1 \\ 1 &{} e^{i2\pi /3} &{} e^{i4\pi /3} \\ 1 &{} e^{i4\pi /3} &{} e^{i8\pi /3} \end{pmatrix}. \end{aligned}$$

In a case where each input of the tritter is injected with biphoton state, the resultant output state after the evolution induced by ${\mathscr {U}}$ is given by

20 $$\begin{aligned} |1,1,1\rangle \xrightarrow {{\mathscr {U}}} \sqrt{\frac{2}{3}}|3,0,0\rangle -\frac{1}{\sqrt{3}}|1,1,1\rangle , \end{aligned}$$

where $|1,1,1\rangle$ corresponds to one biphoton state in each input or output of the tritter, and $|3,0,0\rangle$ represents three biphoton states exiting through one output port and no photons in the other two output ports. The output ports of each tritter are linked with three biphoton mode sorters. At the output of each tritter, the biphoton states from Alice and Bob, are directed towards the Brown Twiss schemes which are tuned to measure the standard basis biphoton modes $|\alpha \rangle$, $|\beta \rangle$ and $|\gamma \rangle$. Each Brown Twiss scheme made up of polarization filters in the arms. The filters comprise a pair of phase-plates and a polarization analyzer used to realize the polarization states of single-photons creating the biphoton. Each Brown Twiss scheme is tuned to detect a certain polarization state of biphoton by setting wave plates ($\lambda /4$ plate, $\lambda /2$ plate) to angle positions that realize desired polarization and setting polarization analyser to allow the desired polarization to pass through. Different angle settings for wave plates are shown in Table 2. When a Brown Twiss scheme is tuned to the settings for detection of specific polarization states, the orthogonal biphoton states cannot result in coincidence detection. For instance, in the Brown Twiss scheme used to detect $|\beta \rangle$ biphoton mode, there are two detectors for the horizontal polarization contributions from Alice and Bob’s biphoton states which are labelled as $D_{HA}$ and $D_{HB}$. Furthermore, there are two detectors for measuring the vertical polarization contributions of biphoton states from Alice and Bob labelled as $D_{VA}$ and $D_{VB}$ as shown in Fig. 3. Similarly, appropriate parameters are set in the filters of both arms of the Brown Twiss scheme for detecting other states to allow coincidence detection. Note that states prepared by Alice and Bob are a superposition of the three basic states $|\alpha \rangle$, $|\beta \rangle$ and $|\gamma \rangle$. Therefore, a successful Bell state measurement in the Brown Twiss scheme corresponds to the observation of precisely 12 detectors being triggered; for instance, $D_{H1A}$, $D_{H1B}$, $D_{H2A}$ and $D_{H2B}$ (associated with $|\alpha \rangle _{L}$ biphoton mode), $D_{H3A}$, $D_{H3B}$, $D_{V4A}$ and $D_{V4B}$ (associated with $|\beta \rangle _{L}$ biphoton mode) and $D_{V5A}$, $D_{V5B}$, $D_{V6A}$ and $D_{V6B}$ (associated with $|\gamma \rangle _{L}$ biphoton mode). These measurement results can be simplified in terms of Bell measurements as $\frac{1}{\sqrt{2}}(D_{\alpha _{L}}D_{\beta _{L}}D_{\gamma _{L}}+D_{\alpha _{R}}D_{\beta _{R}}D_{\gamma _{R}})$ which represent a click in detectors on the left-hand side for $|\alpha \rangle$, $|\beta \rangle$ and $|\gamma \rangle$ modes (i.e.,$D_{\alpha _{L}}$ denotes coincidence detection in detectors of $|\alpha \rangle$ mode) or a click on the right-hand side for $|\alpha \rangle$, $|\beta \rangle$ and $|\gamma \rangle$ modes detectors. A conclusive result is given by click in detectors of the mode sorters on the left-hand side or the right-hand side only. The other Bell state measurements result in inconclusive measurement results, e.g., $\frac{1}{\sqrt{2}}(D_{\alpha _{L}}D_{\beta _{L}}D_{\gamma _{R}}+D_{\alpha _{R}}D_{\beta _{R}}D_{\gamma _{L}})$. The results are provided in Table 3.

**Table 2:** The parameter settings for wave plates in the Brown Twiss schemes.

Wave plate		$|\alpha \rangle$		$|\beta \rangle$		$|\gamma \rangle$
	H	H	H	V	V	V
$\lambda /4$	$-\frac{\pi }{4}$	$-\frac{\pi }{4}$	$-\frac{\pi }{4}$	$-\frac{\pi }{4}$	$-\frac{\pi }{4}$	$-\frac{\pi }{4}$
$\lambda /2$	$\frac{\pi }{8}$	$\frac{\pi }{8}$	$\frac{\pi }{8}$	$-\frac{\pi }{8}$	$-\frac{\pi }{8}$	$-\frac{\pi }{8}$

**Table 3:** The possible Bell state measurement results in the Brown Twiss scheme mode sorting.

States sent by Alice and Bob	Charlie’s measurement results							
	$D_{\alpha _{L}}D_{\beta _{L}}D_{\gamma _{L}}$	$D_{\alpha _{R}}D_{\beta _{R}}D_{\gamma _{R}}$	$D_{\alpha _{L}}D_{\beta _{L}}D_{\gamma _{R}}$	$D_{\alpha _{L}}D_{\beta _{R}}D_{\gamma _{R}}$	$D_{\alpha _{L}}D_{\beta _{R}}D_{\gamma _{L}}$	$D_{\alpha _{R}}D_{\beta _{L}}D_{\gamma _{L}}$	$D_{\alpha _{R}}D_{\beta _{L}}D_{\gamma _{R}}$	$D_{\alpha _{R}}D_{\beta _{R}}D_{\gamma _{L}}$
$|\acute{\alpha }\rangle$ or $|\bar{\alpha }\rangle$	Conclusive	Conclusive	Inconclusive	Inconclusive	Inconclusive	Inconclusive	Inconclusive	Inconclusive
$|\acute{\beta }\rangle$ or $|\bar{\beta }\rangle$	Conclusive	Conclusive	Inconclusive	Inconclusive	Inconclusive	Inconclusive	Inconclusive	Inconclusive
$|\acute{\gamma }\rangle$ or $|\bar{\gamma }\rangle$	Conclusive	Conclusive	Inconclusive	Inconclusive	Inconclusive	Inconclusive	Inconclusive	Inconclusive

### Sifting

Alice and Bob post select states prepared using the same basis when Charlie reports a conclusive event and discards the rest of the data. A conclusive event corresponds to a case where there is a coincidence detection in three Brown Twiss schemes (linked to the same tritter) for three different basis states $|\alpha \rangle$, $|\beta \rangle$ and $|\gamma \rangle$. This occurs when Alice and Bob have prepared the same biphoton state. We define the set ${\mathscr {K}}$ comprising of biphoton signals if Alice and Bob selected the key basis ${\mathscr {M}}_{1}$ and Charlie gets a successful measurement. Similarly, ${\mathscr {C}}$ is a set of post-selected signals from measurement basis ${\mathscr {M}}_{2}$, which are used for monitoring the presence of an adversary. The protocol repeats the first steps until the sifting conditions $|{\mathscr {K}}| \ge n$ and $|{\mathscr {C}}| \ge m$ are met for all *N* signals prepared by Alice and Bob.

### Parameter estimation

The participating parties, Alice and Bob, make use of the random bits obtained from ${\mathscr {K}}$ to create a raw key consisting of bit strings ${\textsf{K}}_{A}$ and ${\textsf{K}}_{B}$. Then, they compute the average error $\frac{1}{|{\mathscr {C}}|} \sum a_{i}\oplus b_{i}$ where $a_{i}$ and $b_{i}$ are Alice and Bob’s bit values.

### Error correction

The information reconciliation scheme is performed, which leaks at least ${\text {leak}}_{EC}$ bits of classical error-correction data. Alice evaluates a bit string (i.e., a hash) measuring length $\log _{2}(1/\varepsilon _{\textrm{cor}})$ using a random ${\text {universal}}_{2}$ hash function to ${\textsf{K}}_{A}$. Then, Alice transmits the choice of the function, including the hash to the receiver, Bob. When the hash of ${\textsf{K}}_{B}$ does not match the hash of ${\textsf{K}}_{A}$, the protocol is aborted.

### Privacy amplification

During this step, Alice uses a random ${\text {universal}}_{2}$ hash function for extracting the length $\ell$ bits of secret key ${\textsf{S}}_{A}$ from ${\textsf{K}}_{A}$. Bob exploits a similar hash function for extracting the key ${\textsf{S}}_{B}$ of length $\ell$ from ${\textsf{K}}_{B}$.

## Security analysis

We consider the realistic scenarios where participating parties exchange finite signals *N* and determine the statistical fluctuations of finite-size key effects. The security analysis follows the proofs provided in Refs.^52–56^ based on a universally composable framework. The protocol creates a pair of key bit strings ${{\textsf{S}}_{{\textsf{A}}}}$ for Alice and ${{\textsf{S}}_{{\textsf{B}}}}$ for Bob. These key bit strings measure length $\ell$ and must satisfy the correctness and secrecy requirements so that the protocol can be considered secure. Based on the composability requirement, the QKD protocol is considered to be correct when ${{\textsf{S}}_{{\textsf{A}}}}={{\textsf{S}}_\mathsf {{B}}}$ for any eavesdropping attack. Thus, the $\varepsilon _{cor}$-correct protocol differs from a correct one according to the error probability, $\varepsilon _{\text {cor}}$, where ${\text {Pr}}[{{\textsf{S}}_{{\textsf{A}}}}\ne {{\textsf{S}}_{{\textsf{B}}}}] \le \varepsilon _{\text {cor}}$. Therefore, the CQ state, $\rho _{{{\textsf{S}}_{\textsf{A}}}E}$ is $\Delta$-secret when

21 $$\begin{aligned} \min _{\rho _E}\frac{1}{2}||\rho _{{{\textsf{S}}_{{\textsf{A}}}}E}-\rho _U\otimes \rho _E||_1\le \Delta , \end{aligned}$$

where $\rho _{{{\textsf{S}}_{{\textsf{A}}}}E}$ is the classical-quantum (CQ) state, which represents the correlation between Alice’s key bit string $S_A$ and Eve’s quantum state $\rho _E$, and $\rho _U$ corresponds to the completely mixed state on the key space.

Since we consider signals generated using spontaneous parametric down-conversion (SPDC) sources that sometimes emit at least one photon pair, our protocol is susceptible to photon number splitting (PNS) attacks. Therefore, we apply the decoy states technique analysis. With the decoy-state approach, Alice prepares photons at random by using the three different intensities $(\mu , \nu , \omega )$ with $\mu$ denoting the signal state intensity, $\nu$ for decoy states, and $\omega$ represents the vacuum states^57^. These intensities are generally chosen according to the following probabilities $P_{\mu }>P_{\nu }>P_{\omega }$ whereby $P_{\mu }, P_{\nu }$, and $P_{\omega }$ represent signal, decoy and vacuum states. Accordingly, signal states attained with the intensity $\mu$ are utilized to generate the final secure key. Finally, the decoy-states acquired based on intensity $\nu$ are used to bound the knowledge of Eve about the key. Therefore, the key rate of the $\varepsilon _{\text {sec}}+\varepsilon _{cor}$-MDI protocol with biphotons is expressed as

22 $$\begin{aligned} \frac{\ell }{N_{\text {total}}}=q\Bigg [Q_{\mu _{a}\mu _{b},{\mathscr {M}}_{1}}^{1,1} (\log _{2}3-h_{3}(e_{{\mathscr {M}}_{2}}^{1,1U}))-{\text {leak}}_{EC}-{\text {log}}_{2}\frac{2}{\varepsilon _{\text {cor}}} -2{\text {log}}_{2}\frac{4}{\varepsilon _{\text {sec}}}\Bigg ], \end{aligned}$$

with $q=\frac{N_{\mu }p_{\kappa }^{2}}{N_{\text {total}}}$, $N_{\mu }$ represents the amount of detected signals prepared according to the intensity $\mu$. In contrast, $p_{\kappa }$ denotes the probability of measuring in the key basis ${\mathscr {M}}_{1}$ and $N_{\text {total}}$ is the total amount of exchanged signals. The term $Q_{\mu _{a}\mu _{b},{\mathscr {M}}_{1}}^{1,1}$ represents the gain of biphotons prepared by Alice and Bob in the key basis ${\mathscr {M}}_{1}$ with intensities $\mu _{a}$ and $\mu _{b}$, respectively. Accordingly, the term $e_{{\mathscr {M}}_{2}}^{1,1U}$ denotes the upper bound on the error rate emanating from single-photon components in the non-key basis ${\mathscr {M}}_{2}$ and ${\text {leak}}_{\textrm{EC}}$ represents the quantity of information leaked to Eve in the error correction step which equals to $N_{\mu }f_{\textrm{EC}}h_{3}(E_{\mu _{a}\mu _{b}{\mathscr {M}}_{1}})$. Here *f* denotes the efficiency due to error correction and $h_{3}(x)$ is the entropy corresponding to three-dimensional quantum states given by $h_{3}(x)=-x{\text {log}}_{2}(\frac{x}{2})-(1-x){\text {log}}_{2}(1-x)$. Finally, the correction terms $\log _{2}(1/\varepsilon _{\textrm{cor}})$ and $2\log _{2}(4/\varepsilon _{\textrm{sec}})$ correspond to the bits of information lost through computation of universal hash function during error correction step and the privacy amplification. The applicable parameters in Eq. (22) are derived in Appendix A.

## Simulation results

We present the analysis of the behavior of the secret key rate in Eq. (22). The simulation results correspond to a fibre-based QKD scheme where the expected key rate is maximized by using the following experimental values where fiber loss coefficient 0.2 dB/km, detector efficiency $\eta =14.5\%$, single-photon detector dark count rate $P_d=1.7\times 10^{-6}$, error correction efficiency $f_{EC}=1.22$, signal states mean photon number $\mu =0.6$ and optimal probability $p_{z}$ for the key basis is 0.95 Ref.^58^.

**Figure 4:**
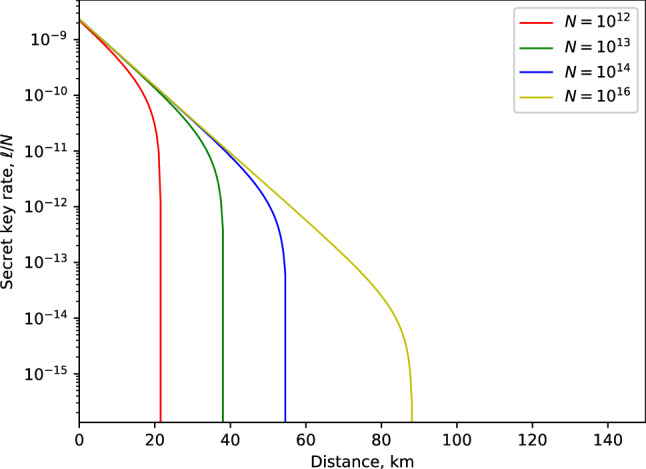
Illustration of the secret key rate (in logarithmic scale) in terms of the transmission distance (km), for a fixed amount of signals $N=10^{12}$, $N=10^{13}$, $N=10^{14}$ and $N=10^{16}$.

**Figure 5:**
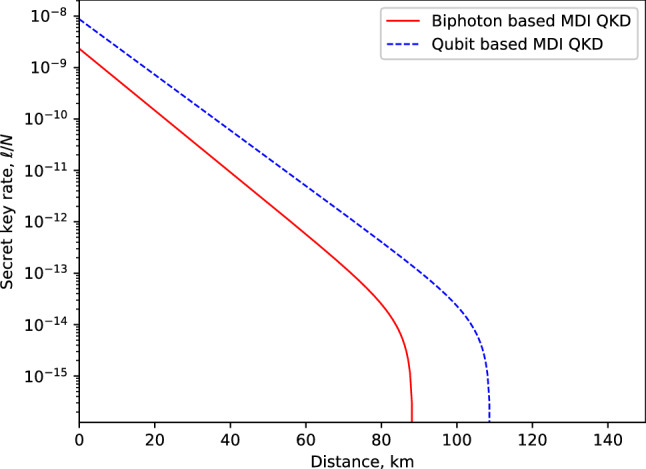
Illustration of the secret key rate (in logarithmic scale) in terms of the transmission distance (km) for our proposed biphoton MDI-QKD and the qubit-based MDI-QKD proposed in Ref.^55^ for a fixed amount of signals $N=10^{16}$.

**Figure 6:**
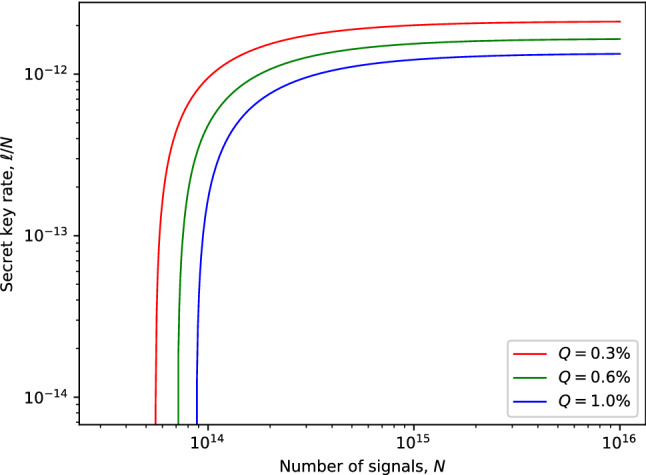
The secret key rate (in logarithmic scale) in terms of the number of signals *N*, for intrinsic misalignment errors $0.3\%$, $0.6\%$, and $1\%$.

Figure 4 depicts the expected secret key rate corresponding to each pulse (i.e., $\ell /N$) in terms of the transmission distance between the participating parties, Alice and Bob, for various amounts of signals *N*. The simulation result demonstrates the feasibility of our proposed protocol in the finite-key regime. Notably, we obtain a fairly reasonable transmission distance of 90 km with realistic $10^{16}$ photon signals. For comparison purposes, we provide the plot for key rate against transmission distance for our proposed biphoton MDI-QKD and the qubit-based MDI-QKD^55^ in Fig. 5. The results indicate that qubit-based MDI-QKD slightly outperforms our proposed biphoton QKD in terms of maximum transmission and achievable key rates. However, it is worth highlighting that the biphoton MDI-QKD provides a higher bit error rate tolerance compared to the qubit-based MDI-QKD. For example, it has previously been demonstrated in Ref.^45,59^ that biphoton-based QKD protocols can tolerate an error rate of about 17.7% to distill a secure key. In contrast, the best qubit-based one-way QKD can only tolerate up to 14.1% in the error rate^45^. Therefore, we highlight that owing to its ability to tolerate a higher quantum bit error rate, the biphoton MDI-QKD can still be considered a reliable candidate for key distribution purposes, particularly over short transmission distances with low losses in the channel. Notably, we remark that the means to create and detect biphoton optical fields have long been successfully investigated^47,60–62^. Furthermore, using comparable schemes the experimental results show that the biphoton states can be realized with high fidelity that ranged from 98.3 % to 99.8%, clearly demonstrating the feasibility of biphoton QKD. Most significantly, the advent of superconducting nanowire single-photon detectors has demonstrated detection efficiency of about 93%^63^. Thus, by harnessing these new technologies, the detection inefficiencies contributed by our detection system, which detectors in our scheme could contribute, can be drastically reduced, resulting in improved key rates. In Fig. 6, we present the performance expected secret key rate (per pulse) $\ell /N$ given as a function of the number of signals *N* for various values of the intrinsic error rate: $Q=(0.3\%,0.6\%, 1\%)$ owing to the misalignment and the optical system’s instability at a distance of 50 km. We show that a minimum data size of about $10^{14}$ signals (attainable current hardware in practical QKD systems) is required to produce a provably secure secret key.

## Conclusion

We presented a high-dimensional QKD protocol employing biphotons to encode information. The biphotons were exploited as qutrits to improve the secret key rate. This is because qutrits carry more bits of classical information and have improved robustness against eavesdropping compared to qubits. Moreover, the secret key rate for the MDI-QKD proposed protocol was simulated regarding the transmission distance for different fixed amounts of signals. These results prove that this protocol achieves a considerable secret key rate for a moderate transmission distance of 90 km by using $10^{16}$ signals. Also, the expected secret key rate was simulated to examine our protocol’s performance at various intrinsic error rate values, $Q=(0.3\%,0.6\%,1\%)$ caused by misalignment and instability due to the optical system. These results show that reasonable key rates are achieved with a minimum data size of about $10^{14}$ signals which are realizable with the current technology. Therefore, the proposed protocol is crucial for realizing practical QKD implementations.

## Data Availability

The datasets generated during and/or analysed during the current study are available from the corresponding author on reasonable request.

## A: Estimation of key rate parameters

We evaluate gains and quantum bit error rates by considering the conditional probabilities for the coincidence detection of *n*-photon pairs in the Brown Twiss scheme. In terms of the basis state $|\alpha \rangle$, the yield is given by

23 $$\begin{aligned} Y_{n,n}^{\alpha }&=[1-(1-Y_{0D_{H1A}})(1-\eta _{D_{H1A}}t)^{n}][1-(1-Y_{0D_{H1B}})(1-\eta _{D_{H1B}}t)^{n}] \\&\times [1-(1-Y_{0D_{H2A}})(1-\eta _{D_{H2A}}t)^{n}][1-(1-Y_{0D_{H2B}})(1-\eta _{D_{H2B}}t)^{n}], \end{aligned}$$

where $Y_{0}$ is the background count rate, $D_{H1A}$, $D_{H1B}$, $D_{H2A}$ and $D_{H2B}$ correspond to detectors for measuring horizontally polarized states in the Brown Twiss scheme. The terms *t* and $\eta$ represent channel transmittance and detection efficiencies. The yield for photon pairs that correspond to state $|\beta \rangle$ is given by

24 $$\begin{aligned} Y_{n,n}^{\beta }&=[1-(1-Y_{0D_{H3A}})(1-\eta _{D_{H3A}}t)^{n}][1-(1-Y_{0D_{H3B}})(1-\eta _{D_{H3B}}t)^{n}] \\&\times [1-(1-Y_{0D_{V4A}})(1-\eta _{D_{V4A}}t)^{n}][1-(1-Y_{0D_{V4B}})(1-\eta _{D_{V4B}}t)^{n}], \end{aligned}$$

where $D_{H3A}$, $D_{H3B}$, $D_{V4A}$, and $D_{V4B}$ correspond to detectors for measuring horizontal and vertical polarized states, respectively, in the Brown Twiss scheme. Similarly, the detection probability for the *n*-photon pair corresponding to $|\gamma \rangle$ is

25 $$\begin{aligned} Y_{n,n}^{\gamma }&=[1-(1-Y_{0D_{V5A}})(1-\eta _{D_{V5A}}t)^{n}][1-(1-Y_{0D_{V5B}})(1-\eta _{D_{V5B}}t)^{n}] \\&\times [1-(1-Y_{0D_{V6A}})(1-\eta _{D_{V6A}}t)^{n}][1-(1-Y_{0D_{V6B}})(1-\eta _{D_{V6B}}t)^{n}], \end{aligned}$$

$D_{V5A}$, $D_{V5B}$, $D_{V6A}$, and $D_{V6B}$ correspond to detectors for measuring vertically polarized states in the Brown Twiss scheme. Therefore, the overall yield is given by

26 $$\begin{aligned} Y_{n,n}=Y_{n,n}^{\alpha }Y_{n,n}^{\beta }Y_{n,n}^{\gamma }. \end{aligned}$$

From these results, the gain for states prepared from key basis comprising of *n*-photon pairs is given by

27 $$\begin{aligned} Q^{n,n}_{\mu _{a}\mu _{b},{\mathscr {M}}_{1}}=Y_{n,n}P(n_{a})P(n_{b}), \end{aligned}$$

where $P(n_{a})$ and $P(n_{b})$ denotes the probabilities for Alice and Bob’s SPDC sources to emit $n_{a}$-photon and $n_{b}$-photon pairs. This probability is expressed as

28 $$\begin{aligned} P(n)=\frac{(n+1)(\frac{\mu }{2})^{n}}{(1+\frac{\mu }{2})^{n+2}}. \end{aligned}$$

The gain corresponding to single-photon contributions is then given by

29 $$\begin{aligned} Q^{1,1}_{\mu _{a}\mu _{b},{\mathscr {M}}_{1}}=\frac{4Y_{1,1}\mu _{a}\mu _{b}}{(2+\mu _{a}+\mu _{b})^{3}}. \end{aligned}$$

In addition, we have the overall gain expressed as

30 $$\begin{aligned} Q_{\mu _{a}\mu _{b},{\mathscr {M}}_{1}}=\sum _{n}^{\infty } Q^{n,n}_{\mu _{a}\mu _{b},{\mathscr {M}}_{1}}, \end{aligned}$$

and the QBER, $E_{\mu _{a}\mu _{b},{\mathscr {M}}_{1}}$ is expressed as

31 $$\begin{aligned} E_{\mu _{a}\mu _{b},{\mathscr {M}}_{1}}Q_{\mu _{a}\mu _{b},{\mathscr {M}}_{1}} =\sum _{n=0}^{\infty }e_{n,n}Q^{n,n}_{\mu _{a}\mu _{b},{\mathscr {M}}_{1}}, \end{aligned}$$

where the error rate of the *n*-photon pairs from Alice and Bob, $e_{n,n}$ is expressed as

32 $$\begin{aligned} e_{n,n}=e_{n,n}^{\alpha }+e_{n,n}^{\beta }+e_{n,n}^{\gamma }. \end{aligned}$$

The error rate for the *n*-photon pair corresponding to the state $|\alpha \rangle$ given by

33 $$\begin{aligned} e_{n,n}^{\alpha }&=[e_{0}(Y_{0D_{H1A}}Y_{0D_{H1B}}+Y_{0D_{H1A}}\eta _{D_{H1B}}+Y_{0D_{H1B}} \eta _{D_{H1A}}+Y_{0D_{H2A}}Y_{0D_{H2B}} \\&\quad +Y_{0D_{H2A}}\eta _{D_{H2B}}+Y_{0D_{H2B}}\eta _{D_{H2A}})+e_{d}(\eta _{D_{H1A}}\eta _{D_{H1B}} +\eta _{D_{H2A}}\eta _{D_{H2B}})]\div Y_{n,n}^{\alpha } \end{aligned}$$

The error rates $e_{n}^{\beta }$ and $e_{n}^{\gamma }$ associated with the detection of states $|\beta \rangle$ and $|\gamma \rangle$, respectively are analogously obtained as

34 $$\begin{aligned} e_{n,n}^{\beta }&=[e_{0}(Y_{0D_{H3A}}Y_{0D_{H3B}}+Y_{0D_{H3A}}\eta _{D_{H3B}} +Y_{0D_{H3B}}\eta _{D_{H3A}}+Y_{0D_{V4A}}Y_{0D_{V4B}} \\&\quad +Y_{0D_{V4A}}\eta _{D_{V4B}}+Y_{0D_{V4B}}\eta _{D_{V4A}})+e_{d}(\eta _{D_{H3A}} \eta _{D_{H3B}}+\eta _{D_{V4A}}\eta _{D_{V4B}})]\div Y_{n,n}^{\beta } \end{aligned}$$

35 $$\begin{aligned} e_{n,n}^{\gamma }&=[e_{0}(Y_{0D_{V5A}}Y_{0D_{V5B}}+Y_{0D_{V5A}}\eta _{D_{V5B}} +Y_{0D_{V5B}}\eta _{D_{V5A}}+Y_{0D_{V6A}}Y_{0D_{V6B}} \\&\quad +Y_{0D_{V6A}}\eta _{D_{V6B}}+Y_{0D_{V6B}}\eta _{D_{V6A}})+e_{d}(\eta _{D_{V5A}} \eta _{D_{V5B}}+\eta _{D_{V6A}}\eta _{D_{V6B}})]\div Y_{n,n}^{\gamma } \end{aligned}$$

Finally, the upper bound on the error rate measured in the complimentary basis used to approximate the phase error rate on the key basis is given by

36 $$\begin{aligned} e_{{\mathscr {M}}_{2}}^{1,1 U}=\frac{E_{\nu _{a}\nu _{b},{\mathscr {M}}_{2}}Q_{\nu _{a}\nu _{b},{\mathscr {M}}_{2}}e^{2\nu }-e_{0}Y_{0}}{Y_{1,1}^{L}\nu ^{2}}, \end{aligned}$$

where

37 $$\begin{aligned} Y_{1,1}^{L}=\frac{P(\mu _{a}|2)P(\mu _{b}|2)[Q_{\nu _{a}\nu _{b},{\mathscr {M}}_{2}} -P(\nu _{a}|0)P(\nu _{b}|0)Y_{0}]-P(\nu _{a}|2)P(\nu _{b}|2)[Q_{\mu _{a}\mu _{b},{\mathscr {M}}_{1}}-P(\mu _{a}|0)P(\mu _{b}|0)Y_{0}]}{P(\mu _{a}|2)P(\mu _{b}|2)P(\nu _{a}|1)P(\nu _{b}|1)-P(\mu _{a}|1)P(\mu _{b}|1)P(\nu _{a}|2)P(\nu _{b}|2)}. \end{aligned}$$

In above equation $P(\lambda |n)=\frac{(n+1)(\frac{\lambda }{2})^{n}}{(1+\frac{\lambda }{2})^{n+2}}$ with $\lambda \in \{\mu , \nu \}$ and *n* denotes number of photons.

## B: Secrecy

Let the system $\tilde{{\textsf{E}}}$ represent information collected by Eve on Alice and Bob’s bit strings ${\textsf{K}}_{A}$ and ${\textsf{K}}_{B}$, respectively, up to the error correction step. By applying the privacy amplification based on the universal class-two hash function, we generate a $\Delta$-secret key of length $\ell$, and

38 $$\begin{aligned} \Delta \le 2\varepsilon +\frac{1}{2}\sqrt{2^{\ell -{\text {H}}_{\text {min}}^{\varepsilon }({\textsf{K}}_{A}|\tilde{{\textsf{E}}})}}, \end{aligned}$$

where ${\text {H}}_{\text {min}}^{\varepsilon }({\textsf{K}}_{A}|\tilde{{\textsf{E}}})$ represents the smooth min-entropy, which corresponds to the average probability which an adversary guesses ${\textsf{K}}_{A}$ correctly using an optimal strategy through having an access to $\tilde{{\textsf{E}}}$. Let $v=\frac{1}{2}\sqrt{2^{\ell -{\text {H}}_{\text {min}}^{\varepsilon }({\textsf{K}}_{A}|\tilde{{\textsf{E}}})}}$, then the secret key length, $\ell$ is given by

39 $$\begin{aligned} \ell ={\text {H}}_{\text {min}}^{\varepsilon }({\textsf{K}}_{A}|\tilde{{\textsf{E}}})-2\log _{2}\Big (\frac{1}{2v}\Big ). \end{aligned}$$

During error correction, Alice and Bob reveal bits of information equals to ${\text {leak}}_{EC}+\log _{2}\Big (\frac{2}{\varepsilon _{\text {cor}}}\Big )$ to an eavesdropper. Therefore, we have that

40 $$\begin{aligned} {\text {H}}_{\text {min}}^{\varepsilon }({\textsf{K}}_{A}|\tilde{{\textsf{E}}})\ge {\text {H}}_{\text {min}}^{\epsilon }({\textsf{K}}_{A}|{\textsf{E}})-{\text {leak}}_{EC}+\log _{2}\Big (\frac{2}{\varepsilon _{\text {cor}}}\Big ), \end{aligned}$$

where ${\textsf{E}}$ is Eve’s information prior to error correction step. Since our analysis is based on decoy states, ${\textsf{K}}_{A}$ can be written in terms of ${\textsf{K}}_{A}^{1}{\textsf{K}}_{A}^{m}$ and this represents the bit strings owing to single photons and multi-photons events. Through using the generalized chain rule for smooth entropies, we have that

41 $$\begin{aligned} {\text {H}}_{\text {min}}^{\epsilon }({\textsf{K}}_{A}|{\textsf{E}})&\ge {\text {H}}_{\text {min}}^{\delta _{1}}({\textsf{K}}_{A}^{1}|{\textsf{K}}_{A}^{m}{\textsf{E}})+{\text {H}}_{\text {min}}^{\delta _{3}}({\textsf{K}}_{A}^{m}|{\textsf{E}})-2\log _{2}\Big (\frac{1}{\delta _{2}}\Big )-1 \\&\ge {\text {H}}_{\text {min}}^{\delta _{1}}({\textsf{K}}_{A}^{1}|{\textsf{K}}_{A}^{m}{\textsf{E}})-2\log _{2}\Big (\frac{1}{\delta _{2}}\Big )-1, \end{aligned}$$

and here the second inequality is based on the fact that ${\text {H}}_{\text {min}}^{\delta _{3}}({\textsf{K}}_{A}^{m}|{\textsf{E}}) \ge 0$. Next, we provide bound for ${\text {H}}_{\text {Twissmin}}^{\delta _{1}}({\textsf{K}}_{A}^{1}|{\textsf{K}}_{A}^{m}{\textsf{E}})$ by using the uncertainty relations for the smooth entropies. To achieve this, we use a gendankenexperiment where Alice and Bob prepare all their states in the basis ${\mathscr {M}}_{2}$ even when they choose the ${\mathscr {M}}_{1}$ basis. In this hypothetical protocol, the bit strings obtained from measurements in the complementary basis, ${\textsf{C}}_{A}$ and ${\textsf{C}}_{B}$ of length $\ell$ replace the keys ${\textsf{K}}_{A}$ and ${\textsf{K}}_{B}$. The smooth min-entropy is expressed as

42 $$\begin{aligned} {\text {H}}_{\text {min}}^{\delta _{1}}({\textsf{K}}_{A}^{1}|{\textsf{K}}_{A}^{m}{\textsf{E}})&\ge n_{1,1\mu }-{\text {H}}_{\text {max}}^{\varepsilon }({\textsf{C}}_{A}|{\textsf{C}}_{B}) \\ {}&=n_{1,1\mu }[1-h(e_{{\mathscr {M}}_{2}}^{1,1})], \end{aligned}$$

where $n_{1,1\mu }=N_{\mu }p_{\kappa }^{2}Q_{\mu _{a}\mu _{b}{\mathscr {M}}_{1}}^{1,1}$ denotes the sifted raw key size obtained from the single photon occurances.

Finally, we combine all the terms which represent errors for min-entropies and error probabilities due to parameter estimation discussed in previous section. Thus, the secrecy is given by

43 $$\begin{aligned} \varepsilon _{\text {sec}}=2\varepsilon +2\delta _{1}+\delta _{2}+v+\varepsilon _{1}+\varepsilon _{2}. \end{aligned}$$

Here, $\varepsilon _{1}$ and $\varepsilon _{2}$ represent error probabilities for estimating the single photon events as well as the phase error rate. The error term is set to a common value $\varepsilon$ and $\varepsilon _{\text {sec}}=8\varepsilon$. The final key length $\ell$ is obtained using the results from Eqs. (40) to (42) and invoking the secrecy requirement from Eqs. (43) in (39). Then, the final derived formula is written as

44 $$\begin{aligned} \ell =N_{\mu }p_{\kappa }^{2}\Bigg [Q_{\mu _{a}\mu _{b},{\mathscr {M}}_{1}}^{1,1} (\log _{2}3-h_{3}(e_{{\mathscr {M}}_{2}}^{1,1U}))-{\text {leak}}_{EC}-{\text {log}}_{2}\frac{2}{\varepsilon _{\text {cor}}}-2{\text {log}}_{2}\frac{4}{\varepsilon _{\text {sec}}}\Bigg ]. \end{aligned}$$
